# Long-Term Feasibility, Adherence, and Exploratory Clinical Outcomes of Crohn’s Disease Exclusion Diet Plus Partial Enteral Nutrition in Adults with Crohn’s Disease

**DOI:** 10.3390/nu18152491

**Published:** 2026-08-02

**Authors:** Ilaria Dileo, Marta Vernero, Davide Giuseppe Ribaldone, Alessia Chiarotto, Anna Avico, Clara Bosa, Marta Ossola, Valentina Ponzo, Gian Paolo Caviglia, Simona Bo

**Affiliations:** 1 Clinical Nutrition and Dietetics Unit, Città della Salute e della Scienza di Torino-Molinette Hospital, 10126 Turin, Italy; idileo@cittadellasalute.to.it (I.D.); achiarotto@cittadellasalute.to.it (A.C.); aavico@cittadellasalute.to.it (A.A.); clbosa@cittadellasalute.to.it (C.B.); mossola@cittadellasalute.to.it (M.O.); simona.bo@unito.it (S.B.); 2 Unit of Gastroenterology, Città della Salute e della Scienza di Torino-Molinette Hospital, 10126 Turin, Italy; mvernero@cittadellasalute.to.it (M.V.); davidegiuseppe.ribaldone@unito.it (D.G.R.); gianpaolo.caviglia@unito.it (G.P.C.); 3 Department of Medical Sciences, University of Torino, 10126 Turin, Italy

**Keywords:** Crohn’s disease, Crohn’s Disease Exclusion Diet, partial enteral nutrition

## Abstract

**Background:** Long-term data on the feasibility, adherence, and efficacy of the Crohn’s Disease Exclusion Diet combined with partial enteral nutrition (CDED + PEN) in adults with active Crohn’s disease (CD) remain limited. **Methods:** In this prospective, single-center cohort study, we assessed adherence, tolerability, clinical remission, nutritional and body composition changes in adults with clinically active CD following a 52-week 3-phase CDED + PEN protocol. Clinical activity, inflammatory markers, quality of life, nutritional intake, and body composition were evaluated at baseline and weeks 6, 12, and 52. **Results:** A total of 22 of 36 patients (61.1%, exact 95% CI:43.5–76.9%) remained adherent throughout the 52-week follow-up. Adherence overlapped with tolerability, as all discontinuations were attributable to poor tolerability. Adherence was higher in patients with disease duration ≤2 years (59.1% vs. 21.4%, *p* = 0.041) and in those with greater caloric (*p* = 0.012) and protein requirements (*p* = 0.022). All adherent patients achieved clinical remission after the 6-week induction phase, and remission was maintained at 12 months in 14/22 (63.6%). Harvey–Bradshaw Index scores decreased significantly at weeks 6 and 12, while fecal calprotectin decreased at week 6. Nutritional status improved, with sustained higher protein intake and early increases in total protein and albumin. Patients maintaining remission at 52 weeks showed greater reductions in C-reactive protein and fecal calprotectin, together with greater improvements in phase angle and muscle mass index. **Conclusions:** CDED + PEN is a feasible and safe long-term strategy for adults with active CD, particularly in early disease. It reduces disease activity, improves nutritional status and body composition, and supports durable remission in a substantial proportion of adherent patients.

## 1. Introduction

Crohn’s disease (CD) is a chronic inflammatory bowel disease of multifactorial etiology, characterized by transmural, segmental inflammation that can involve any part of the gastrointestinal tract [1,2]. Over the past two decades, its management has advanced with the introduction of biologics and small-molecule therapies [3].

Alongside pharmacological progress, increasing attention has focused on environmental factors, particularly diet, in the pathogenesis and modulation of intestinal inflammation [4]. Dietary patterns influence gut microbiota, intestinal permeability, and mucosal immune responses, all central to CD pathophysiology [5,6]. Accordingly, nutritional interventions are emerging as adjunctive therapeutic strategies [7,8].

Dietary approaches such as enteral nutrition and exclusion diets can modulate key pathogenic mechanisms by altering microbiota composition and metabolic activity, reducing exposure to pro-inflammatory luminal factors, and promoting mucosal homeostasis [4,6,8]. This provides a rationale for structured interventions such as the Crohn’s Disease Exclusion Diet (CDED). CDED eliminates foods associated with intestinal inflammation [e.g., processed foods, additives, and certain fats] while emphasizing whole, minimally processed foods, including lean proteins, selected carbohydrates, fruits, and vegetables [9]. It is typically combined with partial enteral nutrition (PEN) [9]. This strategy has shown efficacy in inducing and maintaining remission, particularly in pediatric CD [10], and more recently in adults for both induction and short-term maintenance [11,12]. Real-world studies report remission rates of 50–70% during induction, with sustained remission in a substantial proportion of patients at 6–12 months [12,13,14,15].

These findings have contributed to recent guideline updates, which now include CDED + PEN in selected clinical settings [16,17]. However, despite its favorable safety profile and relevance for patients seeking to avoid corticosteroids, uncertainties remain regarding the long-term tolerance and durability of CDED + PEN. Evidence beyond six months, especially at one year, is limited, and adherence to exclusion diets may be challenging [16,17,18,19,20].

This prospective cohort study therefore aimed to evaluate the long-term feasibility, tolerability, and adherence to the combined CDED + PEN intervention in adults with CD, and to describe exploratory clinical, biochemical, nutritional, and body-composition outcomes over one year.

## 2. Materials and Methods

### 2.1. Study Population

This prospective, single-center cohort study enrolled consecutive adult outpatients with active CD at the IBD Clinic and Clinical Nutrition Unit of AOU Città della Salute e della Scienza, Torino (Italy) between January 2023 and September 2024.

Inclusion criteria were: aged 18–75 years, diagnosis of CD histologically confirmed, active disease at baseline, defined either by a Harvey–Bradshaw Index (HBI) >4 in association with at least one objective marker of inflammation (C-reactive protein [CRP] > 5 mg/L, and/or fecal calprotectin > 250 μg/g), or by evidence of active disease on imaging/endoscopy (Simple Endoscopic Score for Crohn’s Disease [SES-CD] > 2 or bowel ultrasound wall thickness > 3 mm), ability to understand and adhere to the dietary protocol. Exclusion criteria included: symptomatic or obstructive intestinal strictures, undrained intra-abdominal abscesses or complex fistulizing disease requiring immediate surgical intervention, bowel resection within the previous 6 months or planned surgery, initiation or modification of biologic or small-molecule therapies within 3 months prior to enrollment, current use of systemic corticosteroids, pregnancy or breastfeeding, severe comorbidities affecting nutrient absorption, metabolism, or inflammatory status (e.g., chronic renal failure, decompensated liver disease, or short bowel syndrome), concomitant systemic inflammatory conditions, known allergy or intolerance to the components of the polymeric formula or to mandatory CDED foods (e.g., chicken, eggs, potatoes), requirement for specific or restrictive diets for other medical or personal reasons, inability to understand the dietary protocol or to accurately maintain the required 24-h food records, history of or active eating disorders.

### 2.2. Dietary Intervention (CDED + PEN)

Participants were instructed to follow the CDED combined with PEN over 52 weeks. This dietary approach was structured into three consecutive phases (see Supplementary Figure S1):

1. Induction phase [weeks 0 (T0)–6 (T1)]:

CDED: A strict regimen focused on mandatory whole foods (e.g., chicken, eggs, potatoes, fruit) while excluding pro-inflammatory components, including grains, dairy, processed meats, and food additives (e.g., emulsifier, carrageenan);

PEN: Transforming Growth Factor-β2 (TGF-β2)-enriched polymeric formula (Modulen IBD^®^, Nestlé Health Science, Vevey, Switzerland), providing 50% of their total daily energy expenditure (TEE).

2. Early maintenance phase [weeks 7–12 (T2)]:

CDED: gradual reintroduction of a wider variety of whole foods (e.g., selected legumes, additional fruits, and vegetables) while maintaining the exclusion of processed foods and additives;

PEN: the caloric contribution of the polymeric formula was reduced to 25% of the TEE.

3. Long-term maintenance phase [weeks 13–52 (T3)]:

CDED: a “lifestyle” phase allowing further expansion and controlled “free meals” to enhance long-term compliance, while strictly avoiding ultra-processed foods;

PEN: PEN was maintained at 25%.

The intervention was supported by a dedicated multidisciplinary team consisting of two physicians with expertise in clinical nutrition and three registered dietitians, all trained in the management of CD patients. The program was delivered through individual counseling sessions, with written materials and standardized recipes provided to support each phase.

Monitoring included a weekly 24-h dietary record reviewed during monthly in-person visits. Between these evaluations, the dietitians conducted bi-weekly follow-ups (every 14 days) via telephone to assess tolerability and clinical status. Dietary energy and nutrient intakes were assessed through a dietitian-administered 24-h dietary recall at baseline and each of the scheduled follow-up visit. This assessment was distinct from the weekly 24-h dietary records completed by participants and used primarily to monitor adherence to the CDED and PEN prescriptions. The recalls included all foods, beverages, and partial enteral nutrition consumed during the preceding day. Portion sizes were estimated using household measures, standard serving sizes, package information, and a photographic food atlas, when required. Energy and nutrient intakes were calculated using the Italian food composition database developed by the European Institute of Oncology (IEO) [21]. The energy and nutrient contribution of Modulen IBD^®^ was included in the dietary intake calculations. Additionally, participants had continuous access to the clinical nutrition service via telephone or email throughout the 52-week study period to address any questions or concerns regarding the dietary protocol.

### 2.3. Measurements

Clinical, nutritional, and biochemical evaluations were performed at baseline (T0), week 6 (T1), week 12 (T2), and week 52 (T3) (Supplementary Figure S1). All assessments were conducted by two trained gastroenterologists and a dedicated clinical nutrition team.

At each time point of the study (T0, T1, T2, T3) body weight, HBI, and a complete biochemical profile, including complete blood count, CRP, erythrocyte sedimentation rate (ESR), total protein, albumin, and fecal calprotectin, were recorded.

In addition to these, the following were performed:

At Baseline (T0): medical history, CD history, height and calculation of caloric and protein requirements;

At T0, T2, and T3: bioelectrical Impedance Analysis (BIA) and quality of life assessment via the short-form-12 (SF-12) questionnaire;

At T1, T2, and T3: monitoring of adherence to the CDED and PEN protocols.

At the time of enrollment (T0), patients underwent a systematic anamnesis to define demographic data and lifestyle factors. Disease phenotype was classified according to the Montreal Classification [22], specifying disease location (L1–L4). Additionally, any prior intestinal resections, the presence of extra-intestinal manifestations (articular, cutaneous, or ocular), and current pharmacological therapies including aminosalicylates, immunomodulators, prior biologics, steroid in the last year.

Clinical disease activity was quantified using HBI [23]. For the purposes of this study, clinical remission was defined as an HBI score ≤ 4, while clinical response was defined as a reduction in the score of ≥3 points from baseline [23]. Health-related quality of life was evaluated using the validated SF-12 questionnaire [24], from which the Physical Component Summary (PCS) and Mental Component Summary (MCS) scores were derived.

Adherence to the therapeutic intervention was rigorously monitored by assessing compliance with CDED through review of weekly 24-h records, biweekly structured interviews, and a monthly review conducted by an expert clinical dietitian. Adherence with PEN was measured by counting consumed canisters against the prescribed caloric targets.

Anthropometric evaluation included height measurement using a precision mechanical stadiometer (SECA 213) and body weight measurement using a medical electronic column scale (SECA 703, Hamburg, Germany) with 0.1 kg sensitivity for Body Mass Index (BMI) calculation. Body composition was assessed via Bioelectrical Impedance Analysis (BIA) at 50 kHz (Akern BIA 101) under standardized conditions (overnight fast, supine rest). In addition to raw bioelectrical parameters [Resistance (R), height-adjusted Resistance (R/h), Reactance (Xc), height-adjusted Reactance (Xc/h), and Phase Angle (PhA)] the following body composition variables were estimated: total body water (TBW%), extracellular water ratios (ECW/TBW), Muscle Mass Index (MMI = muscle mass/height2), and Fat Mass Index (FMI = fat mass/height2). Bioelectrical impedance analysis was performed at baseline, week 12, and week 52. It was not performed at week 6, as this early assessment was primarily intended to evaluate clinical response, biochemical changes, dietary adherence, and tolerability during the induction phase, whereas body-composition changes were assessed over longer follow-up intervals.

Dietary intakes were evaluated via a 24-h dietary recall. Basal energy expenditure (BEE) and protein requirements were calculated according to ESPEN guidelines [16] using the Harris–Benedict equation, adjusted for total energy expenditure (TEE) based on physical activity levels and disease-induced metabolic stress.

Laboratory investigations were centralized at the laboratory of the “Città della Salute e della Scienza Hospital” of Turin.

Routine hematological and biochemical parameters, including white blood cell (WBC) count, erythrocyte sedimentation rate (ESR), C-reactive protein (CRP), albumin, and total protein, were measured using standard validated assays on automated platforms, according to local laboratory procedures and internal quality-control standards. Fecal calprotectin (FC) was quantified using a fluorescence enzyme immunoassay (FEIA; EliA™ Calprotectin 2, Phadia AB, Uppsala, Sweden).

### 2.4. Definitions

Adherence to the intervention was defined as compliance to more than 90% of the prescribed dietary components and consume at least 90% of the prescribed enteral nutrition volume. Compliance with the CDED is verified based on three fundamental pillars: the daily consumption of mandatory foods (e.g., chicken breast, eggs, potatoes, apples, and bananas), strict adherence to the portions and frequencies of food reintroduced during Phases 2 and 3, and the complete absence of transgressions concerning excluded items such as processed foods, additives (e.g., emulsifiers, carrageenan), gluten, dairy products, and processed meats. Compliance with PEN is defined by consuming an amount of Modulen^®^ corresponding to 50% of TEE during the first 6 weeks and to 25% from week 7 to 52.

Tolerability was defined as the ability to follow the CDED + PEN protocol without significant adverse events or patient-reported intolerance. This included monitoring gastrointestinal symptoms such as nausea, vomiting, abdominal pain or bloating, taste alterations, osmotic diarrhea, or constipation, as well as systemic symptoms including fatigue, headache, dizziness, and dehydration. Factors such as poor palatability or dislike of the diet or enteral formula were also recorded when they result in reduced intake, protocol modification, or discontinuation. 

Clinical remission was defined as a HBI score ≤ 4 without systemic corticosteroids or budesonide. Biochemical remission was defined as the simultaneous normalization of inflammatory markers, namely C-reactive protein ≤ 5 mg/L and fecal calprotectin ≤ 250 µg/g, in patients not receiving systemic corticosteroids or budesonide.

### 2.5. Endpoints

The primary endpoint was long-term adherence to the CDED + PEN protocol at 12 months, defined as completion of the scheduled dietary intervention, including the prescribed PEN component, without discontinuation.

Secondary endpoints included tolerability and safety of the CDED + PEN protocol; exploratory clinical and biochemical outcomes, including clinical remission, clinical response, and biochemical remission at 6 weeks, 12 weeks, and 12 months; changes in BMI, serum albumin, and body-composition parameters assessed by BIA from baseline to 12 months; changes in SF-12 PCS and MCS scores from baseline to 12 months; and need for pharmacological therapeutic escalation, defined as initiation of biologic therapy or corticosteroids.

### 2.6. Ethical Issues

The study was approved by the Ethics Committee of the AOU Città della Salute e della Scienza of Torino (Protocol Number n. 0112390). All participants provided written informed consent prior to being enrolled. All procedures were in line with the Declaration of Helsinki.

### 2.7. Statistical Analysis

Given the prospective, single-arm feasibility design of the study and the limited availability of long-term adult data on CDED + PEN, no formal hypothesis-driven sample size calculation was performed. The sample size was primarily based on the precision of the estimate for the primary endpoint, namely the 12-month adherence rate to the CDED + PEN protocol. Assuming an expected adherence rate of approximately 60%, a sample size of 36 patients would allow estimation of this proportion with a 95% confidence interval half-width of approximately 16 percentage points. This level of precision was considered adequate for the exploratory assessment of long-term feasibility. The study was not powered to detect differences in secondary clinical, biochemical, nutritional, or body-composition outcomes, nor to perform multivariable analyses of factors associated with adherence or remission; therefore, these analyses were considered exploratory.

Patients who discontinued the dietary intervention because of non-adherence were excluded from per-protocol longitudinal analyses after discontinuation. For intention-to-treat analyses, all enrolled patients were included in the denominator, and non-adherent patients requiring therapeutic escalation were classified as non-remitters/treatment failures.

Data distribution was assessed by the Shapiro–Wilk test. Comparisons were performed using Student’s *t*-test for normally distributed continuous variables and the Mann–Whitney test for non-normally distributed variables. Categorical variables were compared using the Chi-square test or Fisher’s exact test, as appropriate. Within-group changes were evaluated by paired Student’s *t*-tests for normally distributed variables and the Wilcoxon signed-rank test for non-normally distributed variables.

Longitudinal changes in continuous outcomes among participants who remained adherent to the intervention were evaluated using linear mixed-effects models fitted by restricted maximum likelihood. Time was included as a categorical fixed effect, and participant was included as a random intercept to account for within-subject correlation. Time was treated categorically because assessments were performed at unequally spaced visits and because non-linear longitudinal patterns were considered plausible. The Kenward–Roger method was used to estimate denominator degrees of freedom and improve small-sample inference. Variables with markedly right-skewed distributions, including erythrocyte sedimentation rate, C-reactive protein, and fecal calprotectin, were natural-log transformed before analysis. When zero values were present, a constant corresponding to half the lowest positive observed value was added before transformation. HBI score was analyzed using a linear mixed-effects model in the primary analysis; the robustness of the findings was assessed using a mixed-effects negative binomial model. The global categorical effect of time was evaluated using the F test from the mixed-effects model and is reported as the overall *p* value for time. This test evaluates whether values differed across study visits and does not represent a test of a linear temporal trend. Post hoc comparisons between each follow-up visit and baseline were adjusted using the Bonferroni method. All tests were two-sided, and a *p* value < 0.05 was considered statistically significant. Statistical analyses were performed using StatSoft STATISTICA, version 12.0 (StatSoft Inc., Tulsa, OK, USA) and, for longitudinal mixed-effects analyses, using StataNow, version 19.5 (StataCorp LLC, College Station, TX, USA).

## 3. Results

### 3.1. Baseline Characteristics and Adherence

Out of 58 patients assessed, 36 satisfied eligibility criteria and were enrolled. Overall, 22 of 36 patients remained adherent to CDED + PEN throughout the 52-week follow-up, corresponding to a 12-month adherence rate of 61.1% (exact 95% CI: 43.5–76.9%).

**Figure 1:**
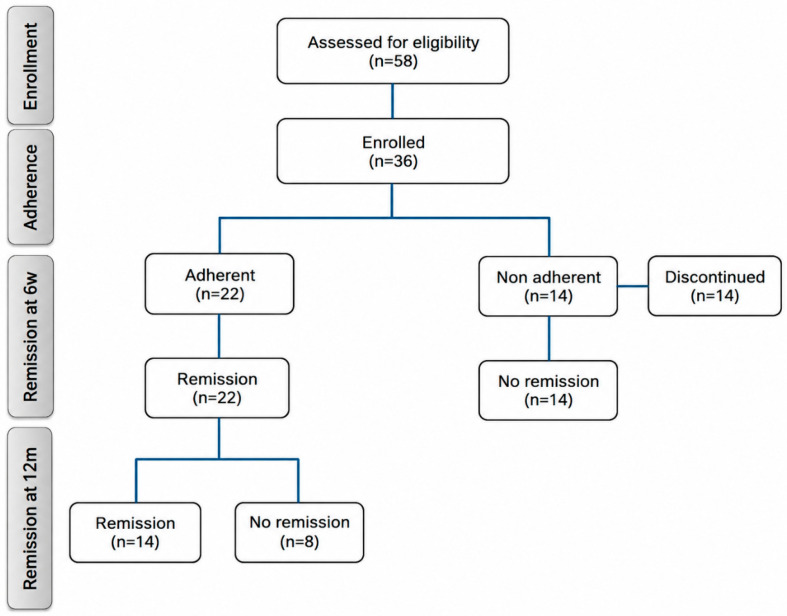
Flow of the study.

Among the 36 enrolled patients, 22 (61.1%) remained adherent to CDED + PEN throughout follow-up, whereas 14 patients (38.9%) discontinued the dietary intervention because of poor tolerance and/or inability to maintain adherence (Figure 1). The main difficulties reported during follow-up were related to adverse event, i.e., nausea (3/14), bloating (1/14), and mild increase in fecal output (6/14), or the restrictiveness of the diet (1/14), acceptability of PEN (1/14), and practical integration of the protocol into daily life (2/14). Non-adherence occurred during the induction phase, within the first 6 weeks of treatment. This phase, characterized by PEN providing 50% of total energy expenditure and the greatest dietary restriction, was the most challenging for adherence.

Adherence was closely correlated with tolerability: indeed, all adherent patients tolerated the treatment. Baseline demographic and clinical characteristics of participants are shown in Table 1.

Onset of the disease within 2 years was significantly more frequent among adherent subjects, while no significant differences were observed between groups for other clinical characteristics. Compared with non-adherent patients, adherent patients showed significantly higher basal and total energy expenditure, greater protein requirements, and higher dietary energy and carbohydrate intake. No significant differences in baseline treatment were observed between the groups, with most patients in both the adherent and non-adherent groups receiving aminosalicylates.

### 3.2. Remission by Adherence

At week 6, all adherent patients achieved clinical remission, whereas none of the non-adherent patients achieved remission (Figure 1). When all enrolled patients were considered according to an intention-to-treat approach, with non-adherent patients classified as non-remitters, the week-6 clinical remission rate was 22/36 (61.1%). Among the 22 adherent patients who achieved remission at week 6, 14 maintained remission at 12 months (63.6%), whereas 8 patients (36.4%) experienced loss of remission during follow-up. In the intention-to-treat population, sustained remission at 12 months was therefore observed in 14/36 patients (38.9%). Among the eight patients who relapsed, relapse occurred after a median of 20 weeks (IQR 12–24) from CDED + PEN initiation. Changes in medical therapy from baseline to 12 months according to adherence status are reported in Supplementary Table S1. Treatment escalation to corticosteroids or biologic therapy was numerically more frequent among non-adherent patients, although the differences between groups were modest.

### 3.3. Changes in Clinical, Nutritional, and Body Composition Parameters in Adherent Patients

Longitudinal changes among the 22 participants who remained adherent to the CDED + PEN intervention were evaluated using mixed-effects models accounting for within-participant correlation (Table 2).

HBI scores showed a significant overall change over time (overall *p* < 0.001). Compared with baseline, HBI scores were significantly lower at weeks 6 and 12 after adjustment for multiple comparisons, whereas the difference at week 52 was no longer statistically significant. These findings were confirmed in the sensitivity analysis using a mixed-effects negative binomial model, which also demonstrated a significant overall time effect (*p* < 0.001), with lower HBI scores at weeks 6 and 12 but not at week 52. The longitudinal evolution of HBI score among adherent patients is shown in Supplementary Figure S2. Body weight showed a modest overall variation over time (overall *p* = 0.021); however, none of the individual follow-up assessments differed significantly from baseline after adjustment for multiple comparisons. No significant overall changes were observed in BMI or in the physical and mental component scores of the SF-12. No significant overall time effects were observed for white blood cell count, erythrocyte sedimentation rate, C-reactive protein, or fecal calprotectin. Fecal calprotectin was significantly lower at week 6 than at baseline in the adjusted post hoc comparison, but the global time effect was not statistically significant. This isolated early difference should therefore be interpreted cautiously. Total protein concentrations showed a significant overall change over time (overall *p* = 0.010), reflecting a transient increase at week 6. Values at weeks 12 and 52 were not significantly different from baseline after adjustment for multiple comparisons. Albumin concentrations also changed significantly over time (overall *p* = 0.002), with significant increases at weeks 6 and 12 but no significant difference from baseline at week 52. Thus, the changes in total protein and albumin were predominantly early and non-linear rather than representing progressive linear trends.

Protein intake increased significantly over time (overall *p* < 0.001) and remained significantly higher than baseline at weeks 6, 12, and 52 after adjustment for multiple comparisons. Although energy intake was numerically higher throughout follow-up, the global effect of time did not reach statistical significance. The isolated significant comparison between week 52 and baseline should therefore be interpreted cautiously in the context of a non-significant global test.

Among body-composition variables, fat mass normalized by height squared decreased significantly over time (overall *p* = 0.031), with a significant reduction at week 12 but not at week 52. Total body water was higher at week 12 in the post hoc comparison, but the global time effect was borderline and did not reach statistical significance (overall *p* = 0.056).

### 3.4. Changes in Clinical, Nutritional, and Body Composition Parameters According to Remission Maintenance

A total of 14 (63.6%) out of 22 adherent patients maintained remission at 12-months of follow-up. Those individuals showed a more favorable clinical and inflammatory profile compared with those who did not maintain remission (Table 3).

At T3, patients maintaining remission continued to demonstrate sustained improvement in clinical disease activity, whereas patients who lost remission experienced a significant worsening in HBI scores. Similarly, maintenance of remission was associated with significant reductions in white blood cell count, C-reactive protein, and fecal calprotectin levels over time, while patients who failed to maintain remission showed either stable or worsening inflammatory markers. A trend toward lower erythrocyte sedimentation rate and higher albumin concentrations was also observed among patients maintaining remission at later follow-up visits.

Patients maintaining remission demonstrated significant improvements in phase angle and muscle mass index at T3, suggesting better preservation of nutritional and cellular status. In contrast, patients who did not maintain remission showed deterioration in phase angle and no improvement in muscle mass. No significant differences were observed between groups in fat mass index or hydration parameters.

Changes in body weight, BMI, quality-of-life scores, total protein levels, energy intake, and protein intake were comparable between groups throughout follow-up.

### 3.5. Adverse Events During Follow-Up in Adherent Patients

No serious adverse events or intervention-related complications were observed during follow-up. No hospitalizations, dehydration requiring medical treatment, or other clinically relevant complications attributable to the CDED + PEN intervention occurred. Among adherent patients, mild transient gastrointestinal symptoms were reported in a minority of cases, including nausea in 4/22 patients and increased bloating in 3/22 patients. These symptoms were self-limiting and did not require discontinuation of the intervention.

## 4. Discussion

In this prospective cohort study, the combined CDED + PEN intervention showed long-term feasibility in a subset of adults with Crohn’s disease. Among patients who remained adherent, CDED + PEN was associated with clinical remission after the induction phase and maintenance of remission at 12 months in a proportion of patients, together with early improvements in fecal calprotectin and favorable changes in nutritional status and body-composition parameters.

### 4.1. Long-Term Adherence to CDED + PEN

Overall, 61.1% of adults with active Crohn’s disease maintained adherence to the CDED + PEN protocol throughout the 52-week follow-up period, whereas approximately 40% discontinued treatment because of poor tolerance to PEN or inability to comply with the dietary restrictions of CDED.

Long-term data on adherence to CDED + PEN remain limited, particularly in adult populations.

A randomized controlled trial including 74 children aged 4–18 years with mild-to-moderate CD compared CDED + PEN with exclusive enteral nutrition (EEN) and found significantly higher tolerance in the CDED + PEN group than in the EEN group (97.5% vs. 73.7%), reflecting the lower rate of treatment discontinuation among patients receiving CDED + PEN [11].

Real-world evidence has also shown that adherence itself may represent a major determinant of treatment success. A retrospective study of 72 patients demonstrated that high adherence to the CDED significantly increases the likelihood of achieving clinical remission at 12 weeks (adjusted OR 7.6; 95% CI 1.07–55.2), confirming the strong link between dietary compliance and therapeutic success [14].

Robust data regarding the long-term adherence and sustainability of the CDED are still lacking, particularly in adults, underscoring the need for prospective studies with extended follow-up. Indeed, a recent systematic review including nine studies and 352 pediatric and adult patients highlighted that, despite encouraging clinical outcomes, the available evidence is largely restricted to short-term follow-up, with most studies reporting outcomes up to 24 weeks [20].

The factors underlying treatment discontinuation remain incompletely understood. In a recent real-world cohort including 69 patients with CD, dietary restrictions, meal planning, social situations, and difficulties eating outside the home emerged as the most common barriers to long-term continuation of the dietary intervention [25].

To our knowledge, the present study represents one of the few prospective real-world experiences evaluating CDED + PEN over 52 weeks in adults with CD.

In exploratory analyses, a disease duration of ≤2 years was associated with greater adherence throughout follow-up. However, because of the limited sample size and the exploratory nature of the study, this finding should be interpreted as hypothesis-generating rather than as evidence of an independent predictor of adherence. This observation raises the possibility that patients in the early phases of disease may be more willing to undertake intensive dietary interventions before exposure to multiple therapeutic failures or prolonged disease burden. Conversely, patients with longer-standing disease may experience greater treatment fatigue, which could reduce their willingness to adhere to a demanding dietary intervention. Finally, non-adherent patients were older than adherent patients, even if this difference was not statistically significant. The observed pattern may reflect greater difficulty in modifying long-established dietary habits or practical challenges related to the complexity of the combined CDED + PEN regimen; however, these hypotheses remain speculative, as the study did not systematically investigate age-related barriers to adherence.

### 4.2. Clinical Remission

All patients who remained adherent to the dietary intervention achieved clinical remission after the 6-week induction phase. However, this finding should be interpreted as a per-protocol result and may be influenced by adherence-related attrition, since patients who did not remain adherent discontinued the intervention and did not achieve remission. When all enrolled patients were considered according to an intention-to-treat approach, with non-adherent patients classified as non-remitters, the week-6 clinical remission rate was 61.1%. At 52 weeks, remission was maintained in 14 of the 22 adherent patients who had achieved remission at week 6, corresponding to 63.6% of adherent patients and 38.9% of the overall enrolled cohort. These findings suggest that the combined CDED + PEN intervention may be associated with early clinical improvement in patients able to follow the protocol, while also highlighting the central role of adherence in determining long-term outcomes. The interpretation of these findings should take into account the limited sample size and the absence of a control group. Therefore, the present results should be considered exploratory clinical outcomes associated with the combined CDED + PEN intervention rather than evidence of definitive effectiveness. Nevertheless, they are relevant because prospective adult data on CDED-based strategies with follow-up extending beyond six months remain limited.

In the randomized controlled trial previously discussed, which included 74 children aged 4–18 years with mild-to-moderate CD, corticosteroid-free remission after 6 weeks was achieved in 75% of patients receiving CDED + PEN compared with 59% of those receiving EEN. Moreover, remission at week 12 was maintained in 75.6% of patients treated with CDED + PEN compared with 45.1% of those initially managed with EEN, supporting the role of dietary exclusion not only in remission induction but also in remission maintenance [11]. The efficacy of CDED + PEN was further supported by a network meta-analysis including 14 randomized controlled trials and 564 pediatric patients. Compared with partial enteral nutrition alone, CDED + PEN was associated with significantly higher rates of clinical remission (OR 7.86, 95% CI 1.85–33.40), while EEN also showed superiority over PEN alone (OR 3.74, 95% CI 1.30–10.76). According to the Surface Under the Cumulative Ranking Curve (SUCRA), a metric used to rank competing interventions in network meta-analyses, CDED + PEN achieved the highest ranking among the dietary strategies included in the analysis for both clinical remission (90.5%) and tolerability (88.0%) [26]. Additional evidence supporting the potential role of CDED + PEN in long-term disease control comes from a retrospective pediatric study including 32 patients with mild-to-moderate CD, in which a Mediterranean-adapted CDED + PEN was compared with EEN. While remission rates were comparable during the induction phase, patients receiving the modified CDED showed significantly higher rates of clinical and biochemical remission at both 12 and 24 months and required biologic therapy less frequently than those treated with EEN (11.1% vs. 50%), suggesting that structured dietary interventions may contribute to sustained disease control beyond remission induction [27].

While these findings provide compelling evidence in pediatric populations, data in adults remain comparatively scarce. In a multicenter prospective open-label trial including 40 adults with mild-to-moderate CD, patients were randomized to receive either CDED + PEN or CDED alone and were followed for 24 weeks. Clinical remission was achieved in approximately 63% of participants after the 6-week induction phase and remained relatively stable throughout follow-up, demonstrating that CDED-based strategies can be successfully implemented in adult populations [13]. Similarly, a prospective study including 32 adults with active CD treated with CDED for 12 weeks reported clinical remission in 76.7% of patients at week 6 and 82.1% at week 12, accompanied by significant reductions in inflammatory markers, further supporting the effectiveness of dietary therapy in routine clinical practice outside randomized trial settings [28]. Taken together, the available literature suggests that CDED-based interventions may support remission induction and maintenance in selected patients with CD. Compared with most adult studies, which have reported outcomes up to 12–24 weeks, the present study provides prospective 52-week observational data and suggests that sustained remission may be achievable in a subset of adults who remain adherent to the combined CDED + PEN intervention. However, because of the small sample size, absence of a control group, and adherence-related attrition, these findings should be interpreted cautiously and require confirmation in controlled studies.

Beyond clinical remission, biochemical remission increased among adherent patients from 36.4% at week 6 to 50.0% at 12 months. This finding is clinically relevant because CRP and fecal calprotectin provide objective, non-invasive measures of inflammatory activity and are commonly used to monitor disease control in Crohn’s disease. The achievement of biochemical remission in half of the adherent cohort at one year suggests possible objective inflammatory improvement over time among patients who were able to sustain the combined intervention. However, longitudinal analyses of the individual inflammatory markers did not show a significant overall effect of time; therefore, the isolated early reduction in fecal calprotectin warrants cautious interpretation.

### 4.3. Impact of the CDED + PEN Protocol on Body Composition

A notable observation of the present study was the change in nutritional and body-composition parameters during follow-up among adherent patients. The sustained increase in dietary protein intake was accompanied by early, non-linear changes in circulating nutritional markers. Total protein increased transiently at week 6, while albumin was higher than baseline at weeks 6 and 12; neither variable differed significantly from baseline at week 52. Body-composition analysis showed a reduction in fat mass index at week 12, while muscle mass index remained substantially unchanged over time. In the overall group of adherent patients, phase angle and extracellular water/total body water ratio also remained stable. However, when patients were stratified according to remission status at 12 months, those who maintained remission showed a more favorable body-composition profile, including greater improvement in muscle mass index and phase angle. Importantly, these changes occurred without significant differences in fat mass index or extracellular hydration parameters, suggesting that the observed differences were not simply attributable to weight gain or fluid shifts, but may reflect a more favorable nutritional and cellular health profile among patients maintaining remission. These findings are particularly relevant in the context of CD, where malnutrition, reduced muscle mass, and sarcopenia are common complications associated with poorer clinical outcomes, increased hospitalization rates, higher postoperative morbidity, and reduced quality of life [29,30]. The improvement in phase angle observed among patients maintaining remission in our study is of particular interest, as phase angle has been proposed as a marker of cellular integrity, nutritional status, and prognosis in inflammatory bowel disease [30].

In the overall adherent cohort, fecal calprotectin decreased significantly during the early phase of the intervention, although the overall effect of time was not statistically significant and no significant differences from baseline were observed at weeks 12 or 52.

However, patients who maintained remission at 12 months showed a more favorable inflammatory profile, characterized by significantly greater reductions in C-reactive protein and fecal calprotectin concentrations than those who relapsed. This may suggest an association between sustained disease control and preservation of nutritional status, although causality cannot be inferred from the present observational design.

To the best of our knowledge, no previous studies have specifically evaluated changes in body composition using bioelectrical impedance analysis during CDED + PEN therapy. Consequently, direct comparisons with previous CDED studies are difficult. In this context, our findings provide preliminary information suggesting that, among adherent patients, the combined CDED + PEN intervention may be associated not only with clinical and inflammatory outcomes, but also with preservation or improvement of muscle mass and nutritional status among patients maintaining remission. These results should be interpreted as exploratory and require confirmation in larger controlled studies.

### 4.4. Clinical Implications

The findings of the present study have several potential clinical implications. First, they suggest that CDED + PEN may be a feasible non-pharmacological strategy in a subset of adults with CD who are able to adhere to the protocol over the long term. However, given the small sample size, absence of a control group, and adherence-related attrition, these findings should be interpreted as exploratory and should not be considered definitive evidence of effectiveness. The observation that patients with more recent disease onset were more likely to remain adherent suggests that dietary therapy may be more acceptable when introduced early in the disease course, although this finding requires confirmation in larger studies.

The favorable changes observed in nutritional status and body composition among adherent patients suggest that the intervention may also be associated with preservation or improvement of muscle mass and nutritional health. This aspect may be clinically relevant given the growing evidence linking reduced muscle mass and sarcopenia to poorer outcomes in patients with CD [31].

Finally, the combined CDED + PEN intervention appeared safe in this cohort, as no serious adverse events or clinically relevant intervention-related complications were observed. Mild transient gastrointestinal symptoms occurred in a small proportion of adherent patients and did not lead to treatment discontinuation.

However, quality-of-life scores did not significantly improve during follow-up, as neither the SF-12 Physical Component Summary nor the Mental Component Summary changed significantly in the overall adherent cohort. This indicates that, despite clinical improvement among adherent patients, the intervention was not associated with a measurable improvement in generic quality of life. The absence of significant change may be related to the small sample size, the demanding nature of the dietary protocol, and the use of a generic quality-of-life instrument, which may be less sensitive than IBD-specific questionnaires in detecting changes related to disease activity, dietary burden, and gastrointestinal symptoms.

Overall, our findings support further investigation of CDED + PEN as part of a personalized and multidisciplinary management strategy for adults with CD, particularly in patients who are motivated and able to adhere to structured dietary therapy.

### 4.5. Strengths and Limitations

The present study has several strengths. First, the prospective design and the 52-week follow-up provide valuable information regarding the long-term feasibility and exploratory clinical outcomes of CDED + PEN, addressing an important gap in the current literature, where most studies have reported outcomes within 6–24 weeks. Second, the study was conducted in a real-world clinical setting, increasing the applicability of the findings to routine practice. Third, the comprehensive assessment of nutritional status, including body composition analysis by bioelectrical impedance analysis, allowed evaluation of outcomes beyond disease activity alone. Finally, participants received intensive multidisciplinary support throughout follow-up, including regular dietetic counseling, scheduled monitoring, and continuous access to the clinical nutrition team, which facilitated the implementation of a complex dietary intervention.

Several limitations should also be acknowledged. The relatively small sample size limits statistical power and may have reduced the ability to identify predictors of long-term adherence and remission maintenance. The single-center design may limit generalizability, and the absence of a control group precludes definitive conclusions regarding causality and the comparative effectiveness of CDED + PEN. Because the study did not include separate CDED-only or PEN-only comparator groups, the independent and relative contribution of each component could not be determined. Therefore, the observed clinical, biochemical, and nutritional changes should be interpreted as outcomes associated with the combined CDED + PEN intervention. Body composition was assessed using BIA, which may be influenced by hydration status and fluid distribution, particularly in patients with active inflammatory disease. Although measurements were performed under standardized conditions, changes in inflammation and hydration during follow-up may have affected impedance-derived estimates, particularly derived parameters such as muscle mass index and fat mass index. Therefore, the observed changes in body composition should be interpreted cautiously. Moreover, endoscopic and transmural healing were not systematically assessed; therefore, the impact of the intervention on objective structural disease outcomes could not be evaluated. Furthermore, adherence was assessed through dietary records and interviews, which are subject to reporting bias. Dietary intake at each assessment point was estimated using a single 24-h dietary recall, which may not fully capture habitual intake or day-to-day variability, particularly if weekday and weekend intake patterns differed.

## 5. Conclusions

In conclusion, the combined CDED + PEN intervention appeared feasible over 12 months in a subset of adults with active CD, particularly among patients with more recent disease onset. Among patients who remained adherent, clinical remission was achieved after the induction phase and maintained in a proportion of cases over 12 months, together with favorable changes in nutritional and body-composition parameters. However, given the small sample size, absence of a control group, and adherence-related attrition, these findings should be interpreted as exploratory and cannot establish definitive effectiveness. Further prospective controlled studies with larger sample sizes are needed to confirm these preliminary findings and to better define factors associated with long-term adherence, sustained remission, and nutritional outcomes in adults undergoing CDED + PEN.

## Figures and Tables

**Table 1:** Baseline characteristics of participants.

	All *n* = 36	Adherent *n* = 22	Non-Adherent *n* = 14	*p*
Age, years	47.2 ± 15.7	43.3 ± 16.2	53.4 ± 13.1	0.057
Males, *n* (%)	17 (47.2)	12 (54.5)	5 (35.7)	0.322
Females, *n* (%)	19 (52.8)	10 (45.5)	9 (64.3)	
Smoking habits, *n* (%)				0.752
Never	14 (38.9)	8 (36.7)	6 (42.8)	
Current	9 (25.0)	5 (22.7)	4 (28.6)	
Former	13 (36.1)	9 (40.9)	4 (28.6)	
Disease duration, years	4.0 (1.0–14.0)	1.5 (0.0–13.0)	7.5 (3.0–19.0)	0.097 *
Onset ≤2 years, n (%)	16 (44.4)	13 (59.1)	3 (21.4)	0.041
Age at diagnosis, years	38.3 ± 16.2	35.1 ± 16.1	43.4 ± 15.7	0.138
Disease location, n (%)				0.825
Ileal	16 (44.4)	10 (45.5)	6 (42.9)	
Ileocolonic	14 (38.9)	9 (40.9)	5 (35.7)	
Colonic	6 (16.7)	3 (13.6)	3 (21.4)	
Prior IBD-related surgery, *n* (%)	7 (19.4)	4 (18.2)	3 (21.4)	0.848
Aminosalicylate use, *n* (%)	28 (77.8)	17 (77.3)	11 (78.6)	1.000
Immunomodulator use, *n* (%)	2 (5.5)	2 (9.1)	0	0.511
Biologic therapy started >3 months before enrolment, *n* (%)	2 (5.5)	0	2 (14.3)	0.144
Steroid use within the last year, *n* (%)	11 (30.6)	8 (36.4)	3 (21.4)	0.564
Extra-intestinal manifestations, *n* (%)	22 (61.1)	13 (59.1)	9 (64.3)	0.969
Physical Component Summary score	37.5 ± 5.0	38.2 ± 5.1	36.5 ± 4.8	0.328
Mental Component Summary score	39.8 ± 6.0	39.4 ± 5.8	40.4 ± 6.4	0.638
Harvey–Bradshaw Index score	6.5 ± 3.1	6.6 ± 3.4	6.2 ± 2.5	0.692
Weight, kg	72.5 ± 13.6	75.9 ± 12.2	67.3 ± 14.4	0.065
Height, cm	166.3 ± 9.2	168.6 ± 9.3	162.6 ± 7.9	0.054
BMI, kg/m^2^	26.4 ± 5.8	27.0 ± 6.0	25.5 ± 5.5	0.444
Body composition				
R, ohm	512.2 ± 67.9	497.4 ± 73.1	535.4 ± 53.1	0.102
R/h, ohm/m	309.5 ± 48.3	296.3 ± 49.7	330.3 ± 39.2	0.038
Xc, ohm	52.7 ± 10.3	51.6 ± 12.1	54.3 ± 6.4	0.458
Xc/h, ohm/m	31.7 ± 6.1	30.6 ± 7.0	33.4 ± 4.1	0.182
PhA, °	5.9 ± 1.1	5.9 ± 1.2	6.0 ± 1.0	0.716
TBW, %	54.6 ± 8.6	54.8 ± 9.0	54.5 ± 8.1	0.916
ECW/TBW, %	47.1 ± 5.2	46.6 ± 5.2	47.9 ± 5.2	0.485
Muscle mass index, kg/m^2^	12.1 ± 2.2	12.6 ± 1.7	11.3 ± 2.8	0.072
Fat mass index, kg/m^2^	8.0 ± 4.5	7.9 ± 4.6	8.2 ± 4.4	0.886
Basal Energy Expenditure, kcal/day	1473.3 ± 192.2	1528.6 ± 205.5	1386.4 ± 133.7	0.028
Total Energy Expenditure, kcal/day	1788.6 ± 273.1	1868.2 ± 302.2	1663.6 ± 160.7	0.012
Protein requirement, g/day	84.5 ± 10.5	87.6 ± 10.1	79.6 ± 9.3	0.022
Nutritional intake				
Energy intake, kcal/day	1670.4 ± 314.2	1753.2 ± 251.8	1540.3 ± 365.5	0.046
Protein intake, g/day	70.4 ± 15.1	72.5 ± 12.4	67.1 ± 18.6	0.306
Protein intake shortfall, g/day	16.0 ± 13.7	16.2 ± 13.5	15.6 ± 14.5	0.891
Fat intake, g/day	65.0 ± 14.6	65.9 ± 14.4	63.6 ± 15.2	0.646
Carbohydrates intake, g/day	198.1 ± 54.0	212.0 ± 43.0	176.1 ± 63.4	0.049
Laboratory variables				
White Blood Cell count, 10^9^/L	7.3 ± 1.5	7.5 ± 1.2	7.1 ± 2.0	0.585
Erythrocyte Sedimentation Rate, mm/h	13.0 (7.0–22.0)	11.0 (7.0–20.0)	13.0 (10.0–27.0)	0.157
C-Reactive Protein, mg/L	1.6 (0.5–5.2)	1.0 (0.4–4.5)	2.6 (1.0–5.4)	0.226
Fecal Calprotectin, μg/g	481.0 (233.0–931.0)	439.5 (233.0–1051.0)	492.0 (290.0–557.0)	0.918
Total protein, g/Dl	6.9 ± 0.7	6.9 ± 0.7	6.9 ± 0.7	0.873
Albumin, g/Dl	4.0 ± 0.5	4.0 ± 0.5	4.2 ± 0.6	0.226

Data are expressed as mean ± SD, median (interquartile range) or n (%). Abbreviations: BMI, body mass index; R, resistance; R/h, height-normalized resistance; Xc, reactance; Xc/h, height-normalized reactance; PhA, phase angle; TBW, total body water; ECW/TBW, extracellular water/total body water ratio; BEE, basal energy expenditure; TEE, total energy expenditure; WBC, white blood cell; ESR, erythrocyte sedimentation rate; CRP, C-reactive protein. * *p*-values calculated by test U Mann–Whitney.

**Table 2:** Longitudinal changes in clinical, biochemical, nutritional, and body-composition variables among adherent participants.

Variables	Baseline *n* = 22	Week 6 *n* = 22	*p* vs. Baseline ^1^	Week 12 *n* = 22	*p* vs. Baseline ^1^	Week 52 *n* = 22	*p* vs. Baseline ^1^	Overall *p* for Time ^2^
Clinical and quality-of-life variables								
Weight, kg	75.9 ± 12.2	74.8 ± 12.1	0.142	74.6 ± 11.6	0.071	76.0 ± 12.3	1.000	0.021
BMI, kg/m^2^	27.0 ± 6.0	26.6 ± 6.0	0.149	27.1 ± 6.0	1.000	27.1 ± 6.0	1.000	0.094
HBI score	6.0 (4.0–10.0)	3.0 (2.0–4.0)	<0.001	3.0 (1.0–6.0)	0.001	3.0 (1.0–12.0)	0.684	<0.001
PCS score	38.2 ± 5.1	38.4 ± 5.7	1.000	40.4 ± 5.6	0.336	37.2 ± 4.6	1.000	0.137
MCS score	39.4 ± 5.8	40.0 ± 7.3	1.000	36.3 ± 7.0	0.340	39.4 ± 7.9	1.000	0.236
Laboratory variables								
White blood cell count, 10^9^/L	7.5 ± 1.2	7.0 ± 1.3	0.572	7.1 ± 1.9	0.908	7.3 ± 1.4	1.000	0.572
ESR, mm/h	11.0 (7.0–20.0)	7.5 (5.0–20.0)	0.324	7.0 (5.0–17.0)	0.060	6.0 (4.0–16.0)	0.061	0.076
CRP, mg/L	1.0 (0.4–4.5)	0.8 (0.3–8.1)	1.000	0.5 (0.2–5.9)	0.535	0.7 (0.2–3.6)	1.000	0.510
Fecal calprotectin, μg/g	439.5 (233.0–1051.0)	241.5 (90.0–535.0)	0.040	238.5 (99.0–846.0)	0.112	243.5 (85.0–720.0)	0.299	0.079
Total protein, g/dL	6.9 ± 0.7	7.2 ± 0.5	0.038	7.1 ± 0.6	0.246	6.9 ± 0.6	1.000	0.010
Albumin, g/dL	4.0 ± 0.5	4.4 ± 0.6	0.001	4.3 ± 0.5	0.018	4.1 ± 0.7	1.000	0.002
Nutritional intake								
Energy intake, kcal/day	1753.2 ± 251.8	1870.7 ± 273.4	0.113	1864.5 ± 252.1	0.147	1890.0 ± 225.3	0.047	0.077
Protein intake, g/day	72.5 ± 12.4	81.1 ± 11.5	<0.001	80.0 ± 10.7	0.002	80.5 ± 9.1	0.001	<0.001
Body composition ^3^								
Resistance, Ω	497.4 ± 73.1	—	—	484.3 ± 53.6	0.172	481.6 ± 71.5	0.076	0.098
Height-normalized resistance, Ω/m	296.3 ± 49.7	—	—	288.7 ± 41.0	0.198	287.0 ± 49.0	0.085	0.110
Reactance, Ω	51.6 ± 12.1	—	—	51.1 ± 10.7	1.000	50.5 ± 11.6	0.581	0.576
Height-normalized reactance, Ω/m	30.6 ± 7.0	—	—	30.3 ± 6.4	1.000	29.9 ± 6.7	0.611	0.594
Phase angle, °	5.9 ± 1.2	—	—	6.0 ± 1.0	0.826	5.9 ± 1.2	1.000	0.688
Total body water, %	54.8 ± 9.0	—	—	56.1 ± 8.7	0.036	55.8 ± 9.0	0.129	0.056
ECW/TBW, %	46.6 ± 5.2	—	—	46.5 ± 4.8	1.000	46.5 ± 5.2	1.000	0.898
Muscle mass index, kg/m^2^	12.6 ± 1.7	—	—	12.7 ± 1.4	1.000	12.6 ± 2.2	1.000	0.840
Fat mass index, kg/m^2^	7.9 ± 4.6	—	—	7.4 ± 4.6	0.014	7.6 ± 4.5	0.141	0.031

Data are presented as mean ± standard deviation or median (interquartile range), as appropriate. Longitudinal changes were evaluated using linear mixed-effects models fitted by restricted maximum likelihood, with time included as a categorical fixed effect and participant included as a random intercept. The Kenward–Roger method was used to estimate denominator degrees of freedom. ESR, CRP, and fecal calprotectin were analyzed after natural-log transformation because of their right-skewed distributions. For CRP and fecal calprotectin, a constant corresponding to half the lowest positive observed value was added before transformation to retain observations with values equal to zero. ^1^ *p* values for model-based comparisons between each follow-up visit and baseline were adjusted using the Bonferroni method. Three comparisons were considered for variables measured at baseline and weeks 6, 12, and 52, and two comparisons for body-composition variables measured only at baseline and weeks 12 and 52. ^2^ Overall *p* values represent the global categorical effect of time obtained from the mixed-effects model and should not be interpreted as tests of a linear trend. ^3^ Body-composition measurements were not performed at week 6. Abbreviations: BMI, body mass index; CRP, C-reactive protein; ECW/TBW, extracellular water-to-total body water ratio; ESR, erythrocyte sedimentation rate; HBI, Harvey–Bradshaw Index; MCS, Mental Component Summary; PCS, Physical Component Summary; TBW, total body water.

**Table 3:** Changes in variables depending on the patient’s ability to maintain (or not maintain) remission.

Variables	Delta T0–T1			Delta T0–T2			Delta T0–T3		
	Maintenance *n* = 14	Non-Maintenance *n* = 8	*p*	Maintenance *n* = 14	Non-Maintenance *n* = 8	*p*	Maintenance *n* = 14	Non-Maintenance *n* = 8	*p*
Weight, Kg	−2.1 (−3.5 +1.0)	−0.5 (−1.6 +1.3)	0.275	−1.2 (−5.0 +1.4)	+0.1 (−1.2 +1.2)	0.275	+0.3 (−2.5 +3.2)	−0.0 (−1.4 +0.5)	0.562
BMI, Kg/m^2^	−0.8 (−1.2 +0.4)	−0.2 (−0.5 +0.4)	0.433	−0.5 (−1.7 +0.9)	0.0 (−0.4 +0.2)	0.453	+0.1 (−0.7 +1.0)	0.0 (−0.5 +0.1)	0.538
HBI score	−3.0 (−4.0 −2.0)	−5.0 (−7.0 −2.5)	0.351	−4.0 (−6.0 −1.5)	−2.5 (−5.0 0.0)	0.190	−4.0 (−6.0 −2.5)	+5.0 (+1.0 +8.5)	<0.001
PCS score	+1.2 (−2.7 +3.4)	−3.6 (−6.5 +7.6)	0.585	+0.6 (−1.0 +6.9)	+0.6 (−4.1 +9.3)	0.838	−0.3 (−3.3 +3.0)	−2.9 (−7.1 −0.4)	0.088
MCS score	−0.5 (−5.4 +4.2)	+1.2 (−1.8 +7.6)	0.633	−3.3 (−8.6 +3.8)	−0.9 (−12.1 +6.4)	0.733	+2.5 (−2.6 +8.1)	−2.0 (−10.7 −0.4)	0.056
White Blood Cell count, 10^9^/L)	−0.7 (−1.4 +0.3)	+0.3 (−0.9 +0.9)	0.125	−1.0 (−2.0 +0.2)	+0.3 (−0.4 +1.8)	0.094	−1.2 (−1.9 −0.5)	+1.0 (+0.5 +2.4)	<0.001
Erythrocyte Sedimentation Rate, mm/h	0.0 (−2.0 +3.0)	−2.5 (−8.5 0.0)	0.281	−0.5 (−2.0 +1.0)	−3.0 (−8.0 0.0)	0.292	−2.5 (−10.0 −1.0)	0.0 (0.0 +0.5)	0.052
C-Reactive Protein, mg/L	−0.1 (−2.0 +0.02)	+0.1 (−0.3 +3.5)	0.138	−0.5 (−1.0 0.0)	+0.2 (0.0 +1.9)	0.040	−0.7 (−3.4 +0.02)	+0.5 (0.0 +3.9)	0.017
Fecal Calprotectin, μg/g	−103.5 (−1050.0 +5.0)	−139.5 (−332.0 +92.5)	0.375	−186.5 (−1050.0 +21.0)	+85.0 (−126.5 +278.0)	0.094	−351.5 (−1130.0 −4.0)	+107.5 (+6.0 +298.5)	<0.001
Total protein, g/dL	+0.2 (0.0 +0.4)	+0.2 (−0.2 +0.4)	0.539	+0.1 (0.0 +0.4)	0.0 (−0.3 +0.5)	0.562	−0.1 (−0.2 +0.3)	−0.1 (−1.0 +0.2)	0.413
Albumin, g/dL	+0.1 (0.0 +1.3)	+0.1 (0.0 +0.8)	0.829	+0.1 (0.0 +0.7)	+0.3 (0.0 +0.7)	0.621	+0.3 (0.0 +0.4)	−0.1 (−0.8 0.0)	0.071
Nutritional intake									
Energy intake, kcal/day	+65.0 (−30.0 +150.0)	0.0 (−122.5 +170.0)	0.581	−5.0 (−120.0 +225.0)	+110.0 (−10.0 +182.5)	0.308	+95.0 (−15.0 +230.0)	+67.5 (−15.0 +155.0)	0.683
Protein intake, g/day	+7.5 (0.0 +25.0)	0.0 (−5.0 +15.0)	0.530	+5.0 (0.0 +20.0)	+2.5 (−5.0 +12.5)	0.580	+5.0 (0.0 +20.0)	+2.5 (−5.0 +15.0)	0.310
Body composition									
R, ohm	-	-	-	−5.0 (−36.0 +6.0)	0.0 (−13.0 +4.5)	0.954	−23.5 (−44.0 +3.0)	0.0 (−10.5 +4.5)	0.221
R/h, ohm/m	-	-	-	−3.0 (−19.7 +3.5)	0.0 (−6.5 +2.7)	0.707	−13.6 (−26.3 +1.3)	0.0 (−5.8 +2.7)	0.161
Xc, ohm	-	-	-	−1.5 (−2.0 +2.0)	0.0 (−1.5 +2.0)	0.641	−1.5 (−4.0 +2.0)	−2.0 (−3.5 +0.5)	0.683
Xc/h, ohm/m	-	-	-	−0.9 (−1.2 +1.0)	0.0 (−0.8 +0.9)	0.562	−0.9 (−2.2 +1.0)	−1.2 (−2.0 +0.2)	0.585
PhA, °	-	-	-	+0.2 (−0.1 +0.5)	0.0 (−0.4 +0.5)	0.749	+0.3 (−0.1 +0.6)	−0.7 (−0.8 −0.2)	0.003
TBW, %	-	-	-	+0.5 (−0.6 +4.2)	+0.6 (−0.6 +1.7)	0.727	+1.9 (−0.9 +3.4)	+0.6 (+0.01 +1.8)	0.954
ECW/TBW, %	-	-	-	+0.4 (−0.9 +1.2)	0.0 (−2.8 +1.6)	0.771	−1.1 (−2.4 +1.2)	+1.0 (−0.3 +2.8)	0.071
Muscle mass index, kg/m^2^	-	-	-	+0.1 (−0.3 +0.6)	0.0 (−0.2 +0.7)	0.973	+0.5 (+0.1 +0.8)	−0.2 (−0.5 0.0)	0.005
Fat mass index, kg/m^2^	-	-	-	−0.1 (−1.3 +0.4)	−0.3 (−0.4 0.0)	0.891	−0.7 (−1.3 +0.4)	−0.1 (−0.3 +0.1)	0.562

Abbreviations: BMI, body mass index; HBI, Harvey–Bradshaw Index; PCS, Physical Component Summary; MCS, Mental Component Summary; WBC, white blood cell; ESR, erythrocyte sedimentation rate; CRP, C-reactive protein; R, resistance; R/h, height-normalized resistance; Xc, reactance; Xc/h, height-normalized reactance; PhA, phase angle; TBW, total body water; ECW/TBW, extracellular water/total body water ratio.

## Data Availability

The data presented in this study are available on request from the corresponding author due to privacy restriction.

## Supplementary Materials

The following supporting information can be downloaded at: https://www.mdpi.com/article/10.3390/nu18152491/s1, Figure S1: CDED + PEN intervention. Figure S2: Evolution of Harvey–Bradshaw Index (HBI) scores across study time points in adherent patients. Table S1: Therapeutic changes during follow-up according to adherence status.

## Abbreviations

The following abbreviations are used in this manuscript:

BEE	Basal energy expenditure
BIA	Bioelectrical impedance analysis
BMI	Body mass index
CD	Crohn’s disease
CDED	Crohn’s Disease Exclusion Diet
CI	Confidence interval
CRP	C-reactive protein
ECW	Extracellular water
ECW/TBW	Extracellular water/total body water ratio
EEN	Exclusive enteral nutrition
ESR	Erythrocyte sedimentation rate
FC	Fecal calprotectin
FEIA	Fluorescence enzyme immunoassay
FMI	Fat mass index
HBI	Harvey–Bradshaw Index
IBD	Inflammatory bowel disease
MCS	Mental Component Summary
MMI	Muscle mass index
OR	Odds ratio
PCS	Physical Component Summary
PEN	Partial enteral nutrition
PhA	Phase angle
R	Resistance
R/h	Height-normalized resistance
SES-CD	Simple Endoscopic Score for Crohn’s Disease
SF-12	12-item Short Form Health Survey
SUCRA	Surface Under the Cumulative Ranking Curve
TBW	Total body water
TEE	Total energy expenditure
TGF-β2	Transforming growth factor-β2
WBC	White blood cell
Xc	Reactance
Xc/h	Height-normalized reactance
