# Supplementary material for: Long-Term Feasibility, Adherence, and Exploratory Clinical Outcomes of Crohn’s Disease Exclusion Diet Plus Partial Enteral Nutrition in Adults with Crohn’s Disease
Source: Nutrients. 2026 Aug 2;18(15):2491. doi: 10.3390/nu18152491 (PMC13468286; doi:10.3390/nu18152491)

Supplementary figure 1. CDED+PEN intervention

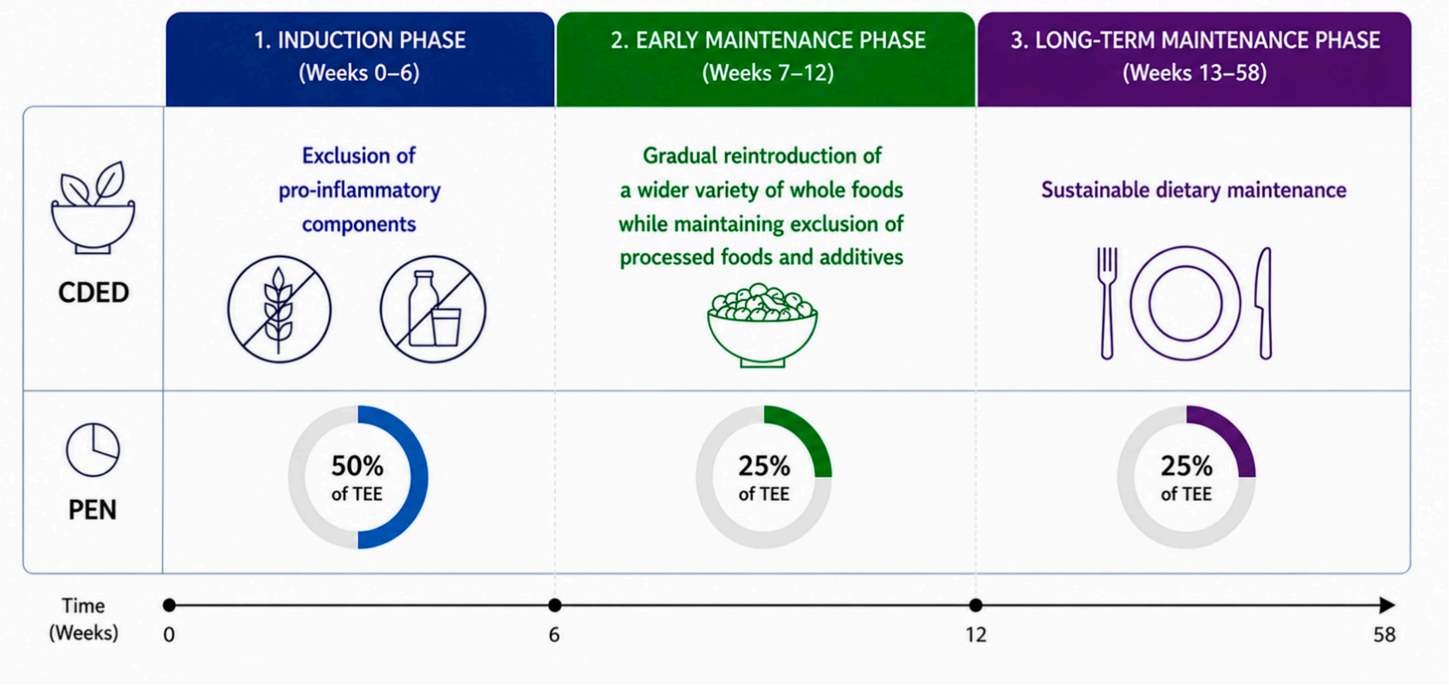

Supplement: Supplementary file 1 [file nutrients-18-02491-s001.zip › supplementary Figure 1 cded+pen.pdf]
