# Supplementary material for: Long-Term Feasibility, Adherence, and Exploratory Clinical Outcomes of Crohn’s Disease Exclusion Diet Plus Partial Enteral Nutrition in Adults with Crohn’s Disease
Source: Nutrients. 2026 Aug 2;18(15):2491. doi: 10.3390/nu18152491 (PMC13468286; doi:10.3390/nu18152491)
Supplement: Supplementary file 1 [file nutrients-18-02491-s001.zip › supplementary table 1.pdf]

Supplementary table 1. Therapeutic changes during follow-up according to adherence status

	Adherent			Non-adherent				
	**Baseline**	**Week 52**	**P within adherent[1]**	**Baseline**	**Week 52**	**P within non-adherent[1]**	**P between groups at baseline[2]**	**P between groups at week 52[2]**
Aminosalicylate use, n (%)	17 (77.3)	17 (77.3)	1.000	11 (78.6)	11 (78.6)	1.000	1.000	1.000
Immunomodulator use, n (%)	2 (9.1)	2 (9.1)	1.000	0 (0.0)	1 (7.1)	1.000	0.511	1.000
Biologic therapy started >3 months before enrolment, n (%)	0 (0.0)	3 (13.6)	0.250	2 (14.3)	5 (35.7)	0.250	0.144	0.217
Systemic corticosteroid or budesonide use, n(%)	0 (0.0)	3 (13.6)	0.250	0 (0.0)	5 (35.7)	0.063	—	0.217

Data are presented as n (%).

[1] Within-group comparisons between baseline and week 52 were performed using the exact McNemar test.

[2] Between-group comparisons at each time point were performed using Fisher’s exact test.
